# Stoichiometric traits (N:P) of understory plants contribute to reductions in plant diversity following long‐term nitrogen addition in subtropical forest

**DOI:** 10.1002/ece3.7319

**Published:** 2021-03-09

**Authors:** Jianping Wu, Fangfang Shen, Jill Thompson, Wenfei Liu, Honglang Duan, Richard D. Bardgett

**Affiliations:** ^1^ Yunnan Key Laboratory of Plant Reproductive Adaptation and Evolutionary Ecology Yunnan University Kunming China; ^2^ Key Laboratory of Soil Ecology and Health in Universities of Yunnan Province College of Ecology and Environmental Sciences Yunnan University Kunming China; ^3^ Jiangxi Key Laboratory for Restoration of Degraded Ecosystems & Watershed Ecohydrology Nanchang Institute of Technology Nanchang China; ^4^ UK Centre for Ecology & Hydrology Penicuik UK; ^5^ Department of Earth and Environmental Sciences The University of Manchester Manchester UK

**Keywords:** ecological stoichiometry, nitrogen deposition, subtropical forest, understory plants, water use efficiency

## Abstract

Nitrogen enrichment is pervasive in forest ecosystems, but its influence on understory plant communities and their stoichiometric characteristics is poorly understood. We hypothesize that when forest is enriched with nitrogen (N), the stoichiometric characteristics of plant species explain changes in understory plant diversity. A 13‐year field experiment was conducted to explore the effects of N addition on foliar carbon (C): N: phosphorus (P) stoichiometry, understory plant species richness, and intrinsic water use efficiency (iWUE) in a subtropical Chinese fir forest. Four levels of N addition were applied: 0, 6, 12, and 24 g m^−2^ year^−1^. Individual plant species were categorized into resistant plants, intermediate resistant plants, and sensitive plants based on their response to nitrogen addition. Results showed that N addition significantly decreased the number of species, genera, and families of herbaceous plants. Foliar N:P ratios were greater in sensitive plants than resistant or intermediate resistant plants, while iWUE showed an opposite trend. However, no relationship was detected between soil available N and foliar N, and soil N:P and foliar N:P ratios. Our results indicated that long‐term N addition decreased the diversity of understory plants in a subtropical forest. Through regulating water use efficiency with N addition, sensitive plants change their N:P stoichiometry and have a higher risk of mortality, while resistant plants maintain a stable N:P stoichiometry, which contributes to their survival. These findings suggest that plant N:P stoichiometry plays an important role in understory plant performance in response to environmental change of N.

## Graphical abstract

We found the N:P stoichiometric traits of understory plants regulate their loss or presence, in which sensitive plants have flexible N:P stoichiometry and higher risk of extinction than resistant plants that have stable N:P stoichiometry to facilitate them living in the N enrichment environments.

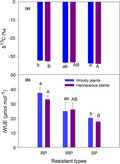

## 1 INTRODUCTION

Over the past century, fossil‐fuel burning and artificial fertilizer application have substantially increased the global nitrogen (N) deposition (Decina et al. 2020; Galloway et al., 2008; IPCC, 2014). For example, N emissions have increased substantially in recent years due to rapid industrialization and urbanization (Liu, 2019; Yu et al., 2019), and an estimated 7.6–20 Tg of reactive N is being emitted into the atmosphere in China (Cui et al. 2013). Much research has focused on understanding how ecosystem processes and plant diversity respond to increasing N deposition (Valliere et al. 2020; Yang et al. 2020; Zak et al. 2019), and many studies show that N deposition poses a significant threat to plant diversity and causes substantial changes in the plant community composition in terrestrial ecosystems (de Vries & Bobbink, 2017; Zhang et al. 2019). Major drivers of such effects of N on plant communities are soil acidification (Lu et al. 2014) and altered plant‐plant interactions (Gilliam et al., 2016; Liu et al. 2017). However, mechanisms explaining reductions in plant diversity in response to long‐term N addition remain unresolved.

Plant elemental allocation, that is, the leaf contents of carbon (C), N, phosphorus (P), and their C:N:P stoichiometric ratios, is highly species specific (Elser et al., 2007; Reich & Oleksyn, 2004; Tian et al., 2018). N enrichment can directly affect plant N allocation via leaf absorption and root uptake (Bobbink et al., 2010), and thus may change leaf N:P stoichiometry (Mayor et al. 2014) and affect ecosystem properties (Huang et al. 2016; Li et al. 2020). Since plant transpiration or water use efficiency regulates plant nutrient supply in response to N deposition (Lu et al., 2018). Further, due to reduced soil P concentration after N addition (Deng et al. 2017; Marklein & Houlton, 2012), N enrichment can affect leaf P concentrations by altering ecosystem P cycling (Marklein & Houlton, 2012). Recent studies show that both below‐ground community of soil microorganisms (Zheng et al. 2019) and above‐ground plant community (Fan et al., 2015) are strongly related to plant N:P stoichiometry, and that plant C:N:P stoichiometric characteristics regulate ecosystem functions such as plant production and carbon cycling (Li et al., 2020). However, the relationships between the stoichiometric N:P ratios and plant species richness under long‐term anthropogenic N deposition are poorly understood.

The aim of this study was to investigate the effects of long‐term N addition on the leaf C:N:P stoichiometric characteristics of the understory plant community in a subtropical forest. We wanted to determine if the responses of plant diversity to N addition are regulated by species‐specific stoichiometry. We hypothesized that: (a) long‐term N addition will negatively impact understory plant diversity; (b) sensitive, resistant, and intermediate resistant plant species will have different stoichiometric responses to long‐term N addition; and (c) reductions in plant diversity resulting from N addition are related to leaf C:N:P stoichiometric characteristics, with sensitive plant species showing greater changes in their stoichiometric characteristics after N enrichment. These hypotheses reflect previous research showing that N enrichment causes changes in plant diversity, stoichiometric characteristics, and water use efficiency in other forest ecosystems (Maes et al., 2020; Maxwell et al., 2019; Valliere et al. 2020). Our study was investigated in a Chinese fir (*Cunninghamia lanceolata*) forest, where we explored responses of understory plants to experimental long‐term (13 years) N addition. The understory plant species were subdivided into three N resistant types based on previous studies, that is, resistant, intermediate resistant, and sensitive plants (Wu et al. 2013). Understory plant diversity, foliar C:N:P stoichiometric characteristics, and intrinsic water use efficiency were also measured in the long‐term field experiment.

## 2 MATERIALS AND METHODS

### 2.1 Study site

The study was conducted in Chinese fir (*C. lanceolata*) plantation forest at the Guanzhuang National Forestry Farm (117°43′E, 26°30′N) in Sanming City, Fujian Province, South China. The ecosystem has a typical subtropical monsoon climate with mean annual precipitation range of 1,606–1,650 mm and a mean annual temperature range of 18.8–19.6°C. Annual average N deposition (including NO_3_^−^ and NH_4_^+^) from rainfall is 4.2–5.7 g N m^−2^ year^−1^ in Sanming region (Xiao, 2005). The soil is an acrisol in the U.S. soil taxonomy (soil organic carbon 18.39 g/kg, soil bulk density 1.06 g/cm^3^, and soil pH 4.68) (Wu et al. 2013). Chinese fir forest was selected for this study because it is widespread in subtropical China and *C. lanceolata* is of high commercial value (Wei et al. 2012). The forest used in this study was planted in 1992 at a density of 1,660 trees per ha^−1^ and covered approximately 5,170 ha on land with uniform site characteristics. At the beginning of the experiment, average tree height was 12 m and mean diameter at breast height (DBH, 1.3 m from the ground) was 16.1 cm. The dominant understory species include *Ardisia punctate*, *Smilax china*, *Arachniodes hasseltii*, and *Ficus hirta*.

### 2.2 Experimental design

The experiment was set up in December 2003 when the *C. lanceolata* plantations were 12 years old. Briefly, 12 plots with 20 × 20 m were randomly established over a 6‐ha section of the plantation. There were four treatments randomly located in each of three replicated blocks. The treatment names and amount of N added in g N m^−2^ year^−1^ are as follows: N0 (control, 0 g N m^−2^ year^−1^), N1 (6 g N m^−2^ year^−1^), N2 (12 g N m^−2^ year^−1^), and N3 (24 g N m^−2^ year^−1^). Urea [CO(NH_2_)_2_] is used as N source in our experiment. For each treatment plot, the required amount of urea [CO(NH_2_)_2_] was dissolved in 20 L of tap water, and the solution was sprayed onto the forest floor within the plots every month starting in January 2004. The control plots (N0) received an equivalent volume of water without CO(NH_2_)_2_.

### 2.3 Plant investigation and sampling analyses

Understory plant communities were investigated in September 2011, 7 years after the start of the N addition treatment (Wu et al. 2013). One 5 × 5 m subplot was established in each 20 × 20 m plot, and all plants taller than 5 cm were recorded. Plant richness (family, genera, and species) and percentage cover were evaluated within each subplot. Understory plant species were divided into two functional groups: woody plants and herbaceous plants. In September 2016 (13 years after N addition started), the understory plants were reassessed using the same methods as in 2011 (Wu et al. 2013). We divided the understory species into three resistance types: resistant plants (RP), intermediate resistant plants (IRP), and sensitive plants (SP) based on the mean presence and absence of the plant species recorded in 2011. Briefly, plant species found in plots with any of the three N addition treatments and in the control (N0) plots, were classified as RP. Plant species found in the N0, N1, and N2 treatment plots, but not in the N3 treatment plots, were categorized as IRP. Finally, plant species found only in N0 treatment plots were categorized as SP.

After the second plant community assessment (species presence and abundance in each treatment plot) in 2016, plant leaves were immediately collected in each plot to measure leaf C, N, P, and their C:N:P stoichiometric traits. Leaves from 22 plant species were collected from the twelve plots of four treatments for foliar chemical analysis. For each individual plant, 5 mature leaves were sampled and bulked to make a composite sample. The fresh plant leaves were oven‐dried at 60°C then ball milled prior to analysis natural abundance of ^13^C, and total C, N, and P. Foliar P was analyzed by persulfate oxidation followed by colorimetric analysis (Bao, 2000), whereas foliar C and N were analyzed by Elemental Analyzer (Flash 200 EA‐HT, Thermo Fisher Scientific, Inc.). Foliar δ^13^C was analyzed by Isotope Ratio Mass Spectrometer (Deata V Advantage, Thermo Fisher Scientific, Inc.). The standards for foliar ^13^C were Pee Dee Belemnite. The calculation as the following:

$$\delta^{13}C=\left(R_{\text{sample}}/R_{\text{standard}}‐1\right)\times 1,000\;\left(‱\right),$$

where *R* represents the isotope ratio (^13^C/^12^C) from samples or standards. The analytical precision for δ^13^C was better than 0.1‰. Based on the value of δ^13^C, we calculated the intrinsic water use efficiency (iWUE) according to the descriptions from Lu et al. (2018). Briefly, ^13^C isotopic discrimination (Δ^13^C) was calculated by (δ^13^C_a_ − δ^13^C_plant_)/(1 + δ^13^C_plant_), where δ^13^C_a_ and δ^13^C_plant_ are the δ^13^C values of atmospheric CO_2_ and plant leaves. The δ^13^C values of atmospheric CO_2_ and the CO_2_ concentrations were cited from the Global Monitoring Division of the Earth System Resrearch Laboratory at Mauna Loa Observatory (www.esrl.noaa.gov/gmd/index.html). According the reference of Farquhar et al. (1982), Δ^13^C = *a* + (*b* − *a*)*C_i_*/*C_a_*, where *a* and *b* refer to the fraction from diffusion through stomata (4.4‰) and the fraction by ribulose‐1,5 bisphosphate carboxylase/oxygenase enzyme during carboxylation (27‰), respectively. And *C_i_*/*C_a_* means the ratio of intercellular to atmospheric CO_2_ concentration. Based on the above equations, iWUE = (*C_a_* − *C_i_*)/1.6.

### 2.4 Statistical analyses

The effect of treatment on foliar stoichiometric characteristics was analyzed using a one‐way analysis of variance (ANOVAs). Repeated measures were used to determine the effects of N addition and sampling year (2011 and 2016) on plant communities. The effects of N addition, plant resistant type, and functional group on foliar C:N:P stoichiometric characteristics were assessed with three‐way ANOVAs. Statistical analyses were performed with R version R 3.3.2 (R Core Team, 2016). Differences were considered significant at the 0.05 level. The relationship between foliar N concentration and P concentration was fitted as: *y* = *ax* + *b*, and correlation coefficients were calculated. All correlations were assessed using regression function in Sigmaplot 12.0 (Systat Software Inc.).

## 3 RESULTS

### 3.1 Understory plant species diversity

**FIGURE 1:**
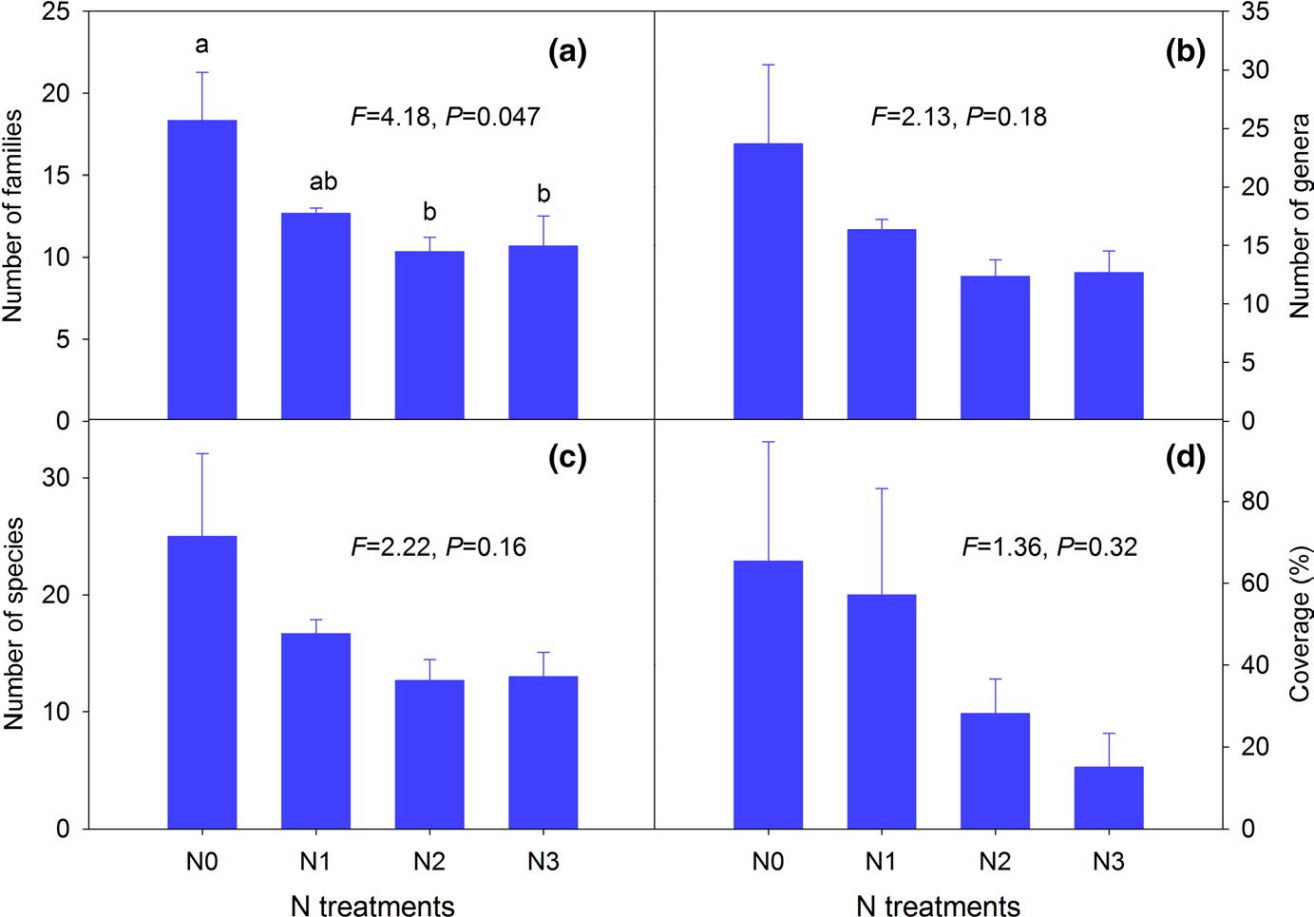
Effects of long‐term N addition on plant richness (species‐1c, genera‐1b, and families‐1a) and percentage plant coverage (1d) of understory plants, under the tree canopy of *Cunninghamia lanceolata* plantation in 2016. Values are means ± *SE*, *n* = 3. N0, N1, N2, and N3 refer to addition of 0, 6, 12, and 24 g of N m^−2^ year^−1^ in each plot, respectively. The statistical effects (*F* and *p* values) of N addition are indicated in figures based on ANOVA and Tukey's honest significant difference test. Different lowercase letters indicate significant differences among N treatments

A total of 68 plant species, 54 genera, and 37 families were found across all plots in 2016. There were 51 species found in N0, 32 species in N1, 28 species in N2 and N3. Long‐term N addition decreased significantly the number of families of understory plants compared with N0 treatment (Figure 1a). The number of genera, species richness, and coverage also showed a declining trend with increasing N addition (Figure 1b–d), although the differences between N treatments were not statistically significant. The combined results for 2011 and 2016 analyzed with a repeated measures ANOVA showed N addition significantly reduced the number of families, genera, and species of understory plants (Table 1). Further, the cover of plant families, genera, and species was decreased by N treatment, although this trend was not significant. There was no interaction for species, genera, or family richness between sampling years (2011 and 2016) and N addition treatments (Table 1).

**TABLE 1:** The effect of long‐term N addition on understory plant species diversity for woody and herbaceous plants in a *Cunninghamia lanceolate* plantation

Factors	*F* (*p*) values					
	Family	Genera	Species	%Coverage	Woody	Herbaceous
SY	0.03 (0.87)	3.82 (0.09)	2,084 (0.13)	3.96 (0.08)	2.17 (0.18)	2.97 (0.12)
N	9.42 (0.005)	9.27 (0.006)	9.50 (0.005)	1.73 (0.24)	1.91 (0.21)	4.18 (0.047)
SY × T	0.45 (0.73)	0.14 (0.94)	0.13 (0.94)	1.19 (0.37)	0.51 (0.69)	0.03 (0.99)

Note

*F* and (*p*) values are given for the effects of repeated measures with sampling year (SY: 2011 and 2016), nitrogen treatments (N), and their interactions, on the plant richness and percentage coverage after long‐term N addition

### 3.2 Foliar C:N:P stoichiometric characteristics

**FIGURE 2:**
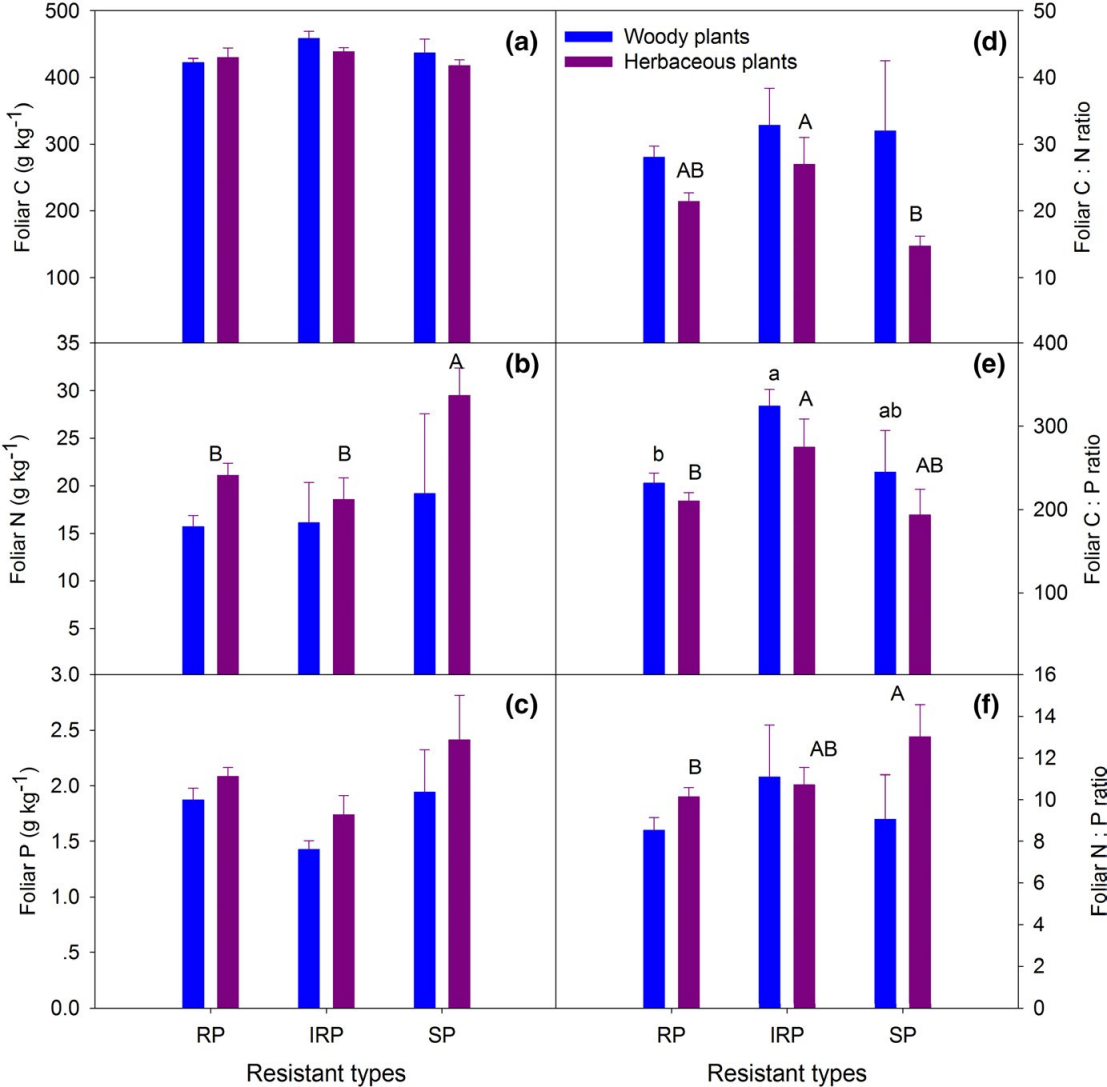
Concentrations (foliar C‐2a, foliar N‐2b, and foliar P‐2c) and stoichiometric characteristics (C:N‐2d, C:P‐2e, and N:P‐2f) of foliar C, N, and P for understory species with different functional groups (woody and herbaceous plants) after long‐term N addition. Plants were also divided into three resistant types, that is, sensitive species (SP), intermediate resistant species (IRP), and resistant species (RP). Values are means ± *SE*, *n* = 3. Within each functional group, means with different letters are significantly different among resistant types based on ANOVA and Tukey's honest significant difference test (*p* <.05). Different lowercase letters indicate significant differences among resistant types for woody plants, different uppercase letters indicate significant differences among resistant types for herbaceous plants

Foliar C did not respond to the N addition treatments (Figure 2a; Table 2), but foliar N was greater for herbaceous plants of the SP than IRP and RP categories (*p* =0.009). Foliar N of herbaceous plants was higher than woody plants within RP group (Figure 2b). Foliar P of SP was slightly higher than for IRP and RP within the herbaceous plant group (*p* =0.066, Figure 2c). The species *S. china* was selected as representative of RP as it was found and sampled in all four (N0, N1, N2, and N3) treatments. Stoichiometric characteristics of foliar C, N, and P for *S. china* did not respond to long‐term N addition (Figure S1).

**TABLE 2:** The effect of long‐term N addition, plant resistant type, and functional group on plant C, N, P, C:N:P stoichiometric characteristics and δ^13^C in a *Cunninghamia lanceolate* plantation

Factors	*F* (*p*) values						
	C	N	P	C:N	C:P	N:P	δ^13^C
N	0.61 (0.66)	0.15 (0.96)	0.04 (0.99)	0.23 (0.92)	0.06 (0.99)	0.15 (0.96)	0.80 (0.53)
RT	0.79 (0.46)	3.83 (0.03)	4.24 (0.02)	2.55 (0.09)	6.93 (<0.01)	2.34 (0.11)	8.74 (<0.01)
FG	0.06 (0.81)	8.07 (0.01)	3.84 (0.06)	11.79 (<0.01)	3.40 (0.07)	3.87 (0.06)	0.55 (0.46)
N × RT	1.43 (0.25)	0.80 (0.46)	0.63 (0.54)	1.07 (0.35)	1.65 (0.21)	2.07 (0.14)	2.38 (0.11)
N × FG	0.61 (0.61)	0.99 (0.41)	1.15 (0.34)	1.14 (0.35)	0.91 (0.45)	1.32 (0.28)	0.90 (0.44)
RT × FG	0.29 (0.75)	0.80 (0.46)	0.25 (0.78)	1.51 (0.24)	0.28 (0.76)	1.57 (0.22)	0.38 (0.69)
N × RT×FG	0.02 (0.90)	1.89 (0.18)	1.10 (0.30)	3.02 (0.09)	1.863 (0.18)	0.83 (0.37)	1.23 (0.27)

Note

*F* and *p* values for the effects of three nitrogen treatments (N), plant resistant type (RT), functional group woody or herbaceous (FG), and their interactions on foliar C, N, P, C:N:P stoichiometric characteristics and δ^13^C after long‐term N addition.

**FIGURE 3:**
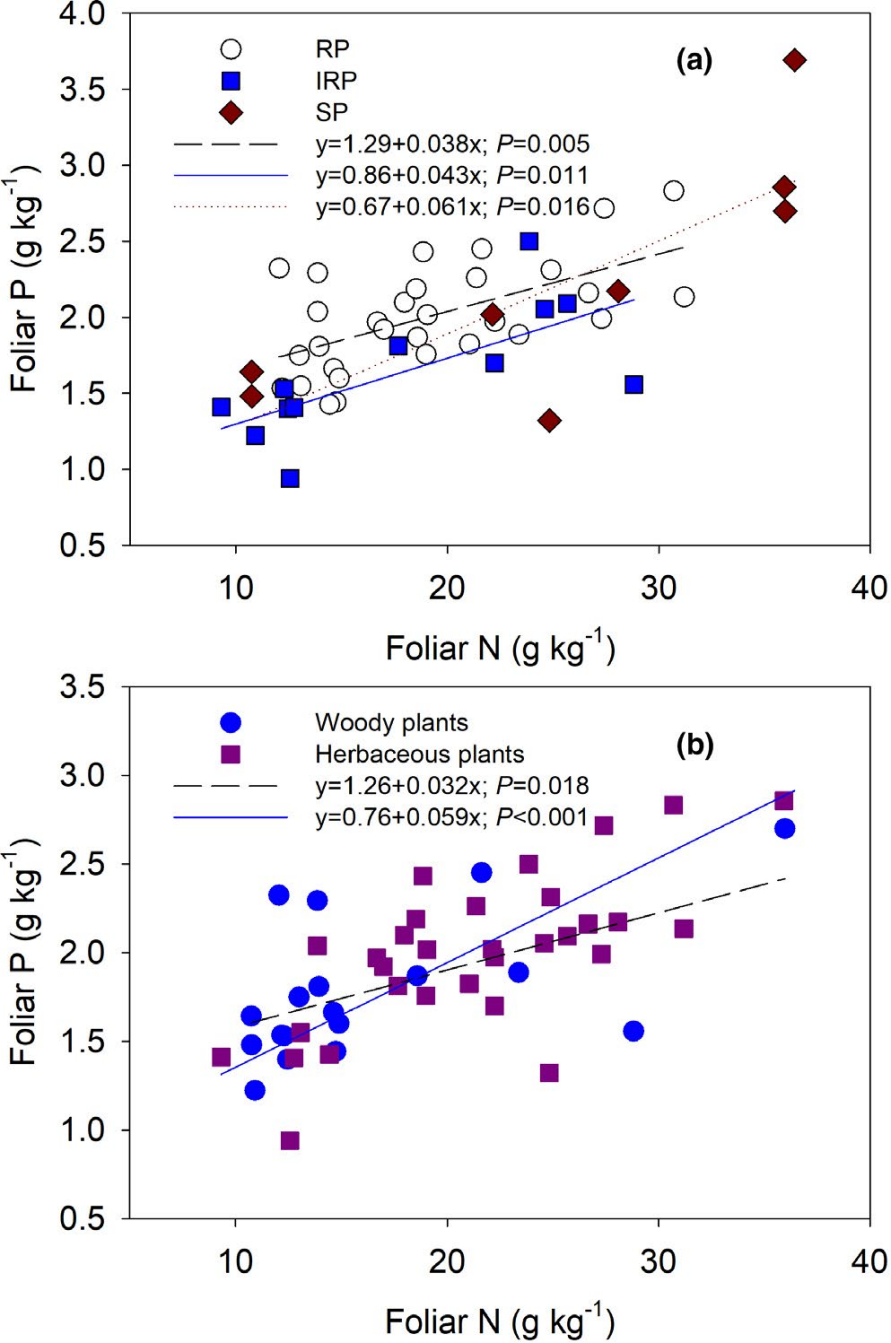
Relationships between foliar N and P concentrations of understory plants with different resistant types (a) and functional groups (b) after long‐term N addition. In the upper (a), open circles with long dash line mean resistant species, solid squares with solid line mean intermediate resistant species, and solid diamonds with short dash line mean sensitive species. In the lower (b), solid circles with solid line mean woody plants and solid squares with long dash line mean herbaceous plants

Within the herbaceous plant group, foliar C:N ratios were lower for SP than IRP and RP plant types (*p* =0.019), but within the woody plant group there was no effect of plant resistant type on foliar C:*N* (Figure 2d). For herbaceous plants, foliar C:N ratios were lower than woody plants within RP and SP groups (*p* =0.003 and *p* =0.072, respectively, Figure 2d). Within both herbaceous (*p* =0.018) and woody plant groups (*p* =0.041, Figure 2e), foliar C:P ratios were significantly lower for SP than IRP and RP. Within the herbaceous plant group foliar, N:P ratios were greatest in SP and were higher for herbaceous plants than woody plants (Figure 2f). For the species resistant types, foliar *N* (*p* =0.03), P (*p* =0.02) and C:P ratio (*p* =0.002) were differed significantly (Table 2). Foliar N and P concentrations were related significantly (Figure 3), but the regression slope of relationship was greater for SP than IRP and RP plant types (Figure 3a), and for herbaceous than woody plants (Figure 3b). No relationship was detected between soil available N and foliar N, or soil N:P and foliar N:P (Figure S2).

### 3.3 Foliar δ^13^C and iWUE

**FIGURE 4:**
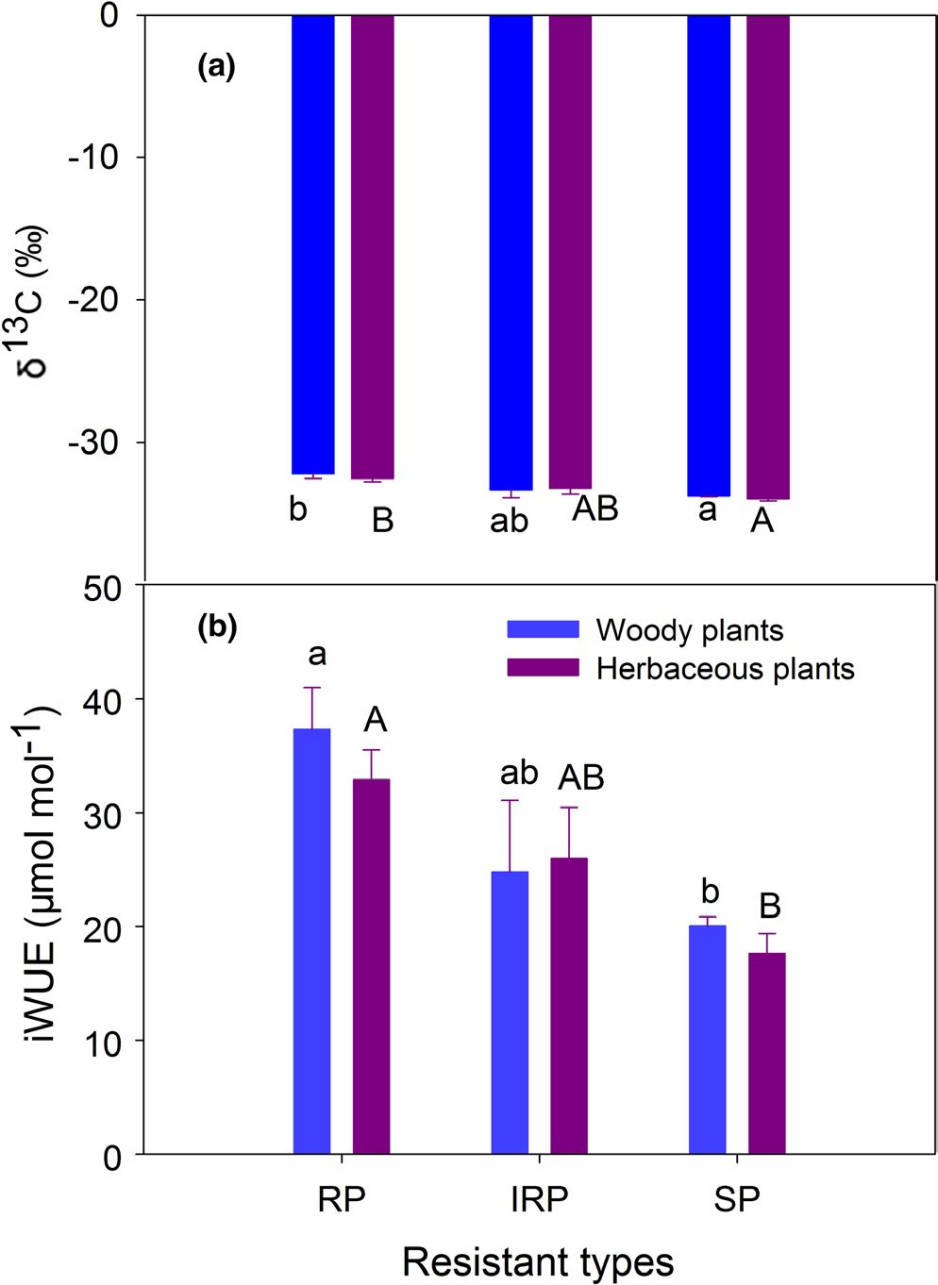
Foliar δ^13^C (a) and intrinsic water use efficiency (iWUE, b) for understory species with different functional groups (woody and herbaceous plants) after long‐term N addition. Values are means ± *SE*, *n* = 3. See Figure 2 for abbreviations

Foliar δ^13^C was highest for the RP plant type and was differed significantly for both woody and herbaceous plant groups (*p* =0.05 and *p* =0.017, respectively). There was no effect of functional group on foliar δ^13^C within the same plant resistant type (Figure 4a). The two‐way ANOVA showed that plant resistant types, but not N treatment, functional group, or their interactions, significantly affected foliar δ^13^C (Table 2). iWUE showed the similar trend with δ^13^C values (Figure 4b). For *S. china*, foliar δ^13^C were from −32.2‰ to −33.8‰ without significant variation among N addition treatments (Figure S3).

## 4 DISCUSSION

Tropical and subtropical forests are at risk from increasing levels of N deposition (de Vries & Bobbink, 2017; Yu et al., 2019), and previous studies have suggested that increasing N deposition reduces plant diversity (Lu et al., 2018; Wu et al. 2013). We conducted a field experiment in a subtropical Chinese fir (*C. lanceolata*) forest to examine long‐term N addition on understory plant diversity and investigate if species water use efficiency influences their responses to N deposition. Consistent with the results of Wu et al. (2013) using data from these plots collected in 2011, and our first hypothesis, N addition negatively affected plant diversity, causing a loss of 33.3%–49.3% of plant species richness during 13 years of N addition treatment. Since the light regime was similar in each plot (Wu et al. 2013), we do not believe the effect of canopy structure on understory shade was a main driver of species distribution. A reduction in plant richness after N addition has been reported for other ecosystems such as grassland (Liu et al. 2017), shrubland (Valliere et al. 2020), and forest (Gilliam, 2019). The potential mechanisms for the decline of plant diversity with N enrichment are widely reported to include factors such as soil acidification, increased aluminium mobility, and increased plant competition from fast‐growing, exploitive species (Bobbink et al., 2010; Gilliam, 2019; Lu et al. 2010). However, our results for this subtropical plantation forest suggest a potential mechanism for the survival of understory plant diversity under N enrichment, namely differences in plant species N:P stoichiometric characteristics. Plants N:P stoichiometry in understory plants showed significant differences among species categorized by presence and abundance in the plots into N resistance types.

Few studies have considered plant N:P stoichiometry as a potential mechanism for the survival of plants in response to N addition, which is surprising given the important role that foliar N:P ratios play in regulating plant production and community dynamics (Tian et al., 2018). Consistent with our second hypothesis, foliar N:P stoichiometry of the different N resistant types responded differently to 13 years of N addition. The category of plant resistance type was the main distinguishing feature reflecting foliar N, P, and N:P stoichiometric characteristics, while the N addition treatment was not statistically significant. In particular, we found that resistant plant type showed negligible response of foliar N and P and their N:P stoichiometric characteristics to long‐term N addition, which we suggest these understory plants to persist under high N inputs. In contrast, the sensitive plant type (SP) had greater leaf concentrations of N than resistant (i.e., IRP and RP plant types) species, which may have induced significant changes in N:P values and increased their vulnerability to loss under elevated N. This is consistent with the notion that plants with plastic stoichiometry can change their intrinsic elemental balance, which increases their risk of loss when experiencing environmental change (e.g., N deposition) (Sterner & Elser, 2002). Further support for the notion that plant stoichiometry underlies plant responses to N addition comes from a recent report that responses of foliar N of dominant understory species were marginal after 8 years of N addition, which can be categorized as resistant plant type (Zou et al. 2019). In addition, a study across nine terrestrial ecosystems in North America demonstrated that both the abundance‐ and functional trait‐based mechanisms explained plant diversity losses to N fertilization (Suding et al., 2005). In our study, insignificant relationships between soil available N and foliar N, or soil N:P and foliar N:P supported the idea that plant stoichiometric traits may play important roles in the response of plants to N addition. Since plant N:P values do not always reflect soil nutrient values, and this was also found in other studies (Deyn, 2017).

Differences in foliar δ^13^C values have been related to water use efficiency and associated photosynthetic capacity (Palmroth et al. 2014). For understory species of N sensitive (SP) plants, foliar δ^13^C was lower than resistant plant (RP) type, which may reflect the lower water use efficiency (Townsend et al. 2007). Foliar δ^13^C in our study of a representative N resistant species (*S. china*) did not significantly respond to long‐term N addition. The resistant species may have higher water use efficiency as indicated by the values of intrinsic water use efficiency in this study, which can reduce water uptake during growth. Consequently, resistant species can reduce N nutrient uptake from soil to plant and facilitate them maintaining stable N:P stoichiometry after N enrichment as predicted by our third hypothesis. In support of this, past study has reported that tropical forests with high N background would acclimate N deposition during their evolutionary history (Lu et al., 2018). Our results were also partially supported the Stability of Limiting Elements Hypothesis (Han et al. 2011), where limiting elements in plants have low variability and environmental sensitivity. Since the resistant plants have more stable N:P stoichiometric characteristics and may be less sensitive to environmental gradients, which helps them survival in N loading sites. In tropical and subtropical forests, N enrichment affects N and P concentrations in plants (Chen et al. 2015; Homeier et al., 2012) and shows species‐specific traits (Huang et al. 2016; Lu et al., 2018). Our findings suggest that decreased understory plant diversity is related to plant N:P stoichiometric characteristics.

## 5 CONCLUSIONS

Our long‐term experimental results have several implications. First, we confirm that long‐term N deposition can decrease plant diversity in subtropical plantation forests. Understory plants are important components of forest ecosystems and the consequence of losing plant diversity needs to be considered under global change phenomenon (e.g. increasing N deposition). Second, our results indicate that the changeable stoichiometry of sensitive plants, that also contain higher N concentration, may result in an elemental imbalance and consequentially put sensitive plants at a higher risk of loss. In contrast, resistant plant types that do not respond to N addition display stable N:P stoichiometric characteristics that enable them to thrive in an environment that may be affected by N deposition. Overall, greater biodiversity is important for ecosystem functions (Landuyt et al., 2019; Mori, 2017), the effects of decreased plant diversity after N deposition on the performance of ecosystem functions need more investigations in the forest ecosystems.

## CONFLICT OF INTEREST

We declare no conflict of interest.

## AUTHOR CONTRIBUTIONS

**Jianping Wu:** Conceptualization (equal); data curation (equal); formal analysis (equal); funding acquisition (equal); investigation (equal); methodology (equal); project administration (equal); resources (equal); software (equal); validation (equal); visualization (equal); writing – original draft (equal); writing – review and editing (equal). **Fangfang Shen:** Data curation (equal); formal analysis (equal); investigation (equal); methodology (equal); writing – original draft (equal). **Jill Thompson:** Conceptualization (equal); formal analysis (equal); methodology (equal); software (equal); supervision (equal); writing – original draft (equal); writing – review and editing (equal). **Wenfei Liu:** Data curation (equal); formal analysis (equal); investigation (equal); project administration (equal); resources (equal); writing – original draft (equal). **Honglang Duan:** Data curation (equal); formal analysis (equal); software (equal); writing – original draft (equal). **Richard D. Bardgett:** Conceptualization (equal); data curation (equal); formal analysis (equal); supervision (equal); writing – original draft (equal); writing – review and editing (equal).

## Supporting information


**Supplementary Figure 1.** Stoichiometric characteristics of foliar C, N and P for Smilax china after long‐term N addition. Values are the means ± SE of three plots. N0, N1, N2, and N3 refer to addition of 0, 6, 12, and 24 g of N m‐2 yr‐1 in each plot, respectively. The statistical effects (F and P values) of N addition were indicated in figures based on ANOVA and Tukey’s honest significant difference test.
**Supplementary Figure 2.** The regressions between soil available N and foliar N (upper), and soil N:P and foliar N:P (lower) after long‐term N addition.
**Supplementary Figure 3.** Foliar δ13C for Smilax china after long‐term N addition. Values are the means ± SE of three plots. N0, N1, N2, and N3 refer to addition of 0, 6, 12, and 24 g of N m‐2 yr‐1 in each plot, respectively. The statistical effects (F and P values) of N addition were indicated in figures based on ANOVA and Tukey’s honest significant difference test.

## Data Availability

Data available from the Dryad Digital Repository: Wu et al. (2021), Dryad, Dataset, https://doi.org/10.5061/dryad.z08kprrb2.
