# Supplementary material for: Stoichiometric traits (N:P) of understory plants contribute to reductions in plant diversity following long‐term nitrogen addition in subtropical forest
Source: Ecol Evol. 2021 Mar 9;11(9):4243–51. doi: 10.1002/ece3.7319 (PMC8093670; doi:10.1002/ece3.7319)
Supplement: Supplementary file 1 — Supplementary Figure 1. Stoichiometric characteristics of foliar C, N and P for Smilax china after long‐term N addition. Values are the means ± SE of three plots. N0, N1, N2, and N3 refer to addition of 0, 6, 12, and 24 g of N m‐2 yr‐1 in each plot, respectively. The statistical effects (F and P values) of N addition were indicated in figures based on ANOVA and Tukey’s honest significant difference test. Supplementary Figure 2. The regressions between soil available N and foliar N (upper), and soil N:P and foliar N:P (lower) after long‐term N addition. Supplementary Figure 3. Foliar δ13C for Smilax china after long‐term N addition. Values are the means ± SE of three plots. N0, N1, N2, and N3 refer to addition of 0, 6, 12, and 24 g of N m‐2 yr‐1 in each plot, respectively. The statistical effects (F and P values) of N addition were indicated in figures based on ANOVA and Tukey’s honest significant difference test. [file ECE3-11-4243-s001.doc]

**Supplementary figure legends**

**Supplementary Figure 1.** Stoichiometric characteristics of foliar C, N and P for *Smilax china* after long-term N addition. Values are the means ± SE of three plots. N0, N1, N2, and N3 refer to addition of 0, 6, 12, and 24 g of N m^-2^ yr^-1^ in each plot, respectively. The statistical effects (*F* and *P* values) of N addition were indicated in figures based on ANOVA and Tukey’s honest significant difference test.

**Supplementary Figure 2.** The regressions between soil available N and foliar N (upper), and soil N:P and foliar N:P (lower) after long-term N addition.

**Supplementary Figure 3.** Foliar δ^13^C for *Smilax china* after long-term N addition. Values are the means ± SE of three plots. N0, N1, N2, and N3 refer to addition of 0, 6, 12, and 24 g of N m^-2^ yr^-1^ in each plot, respectively. The statistical effects (*F* and *P* values) of N addition were indicated in figures based on ANOVA and Tukey’s honest significant difference test.


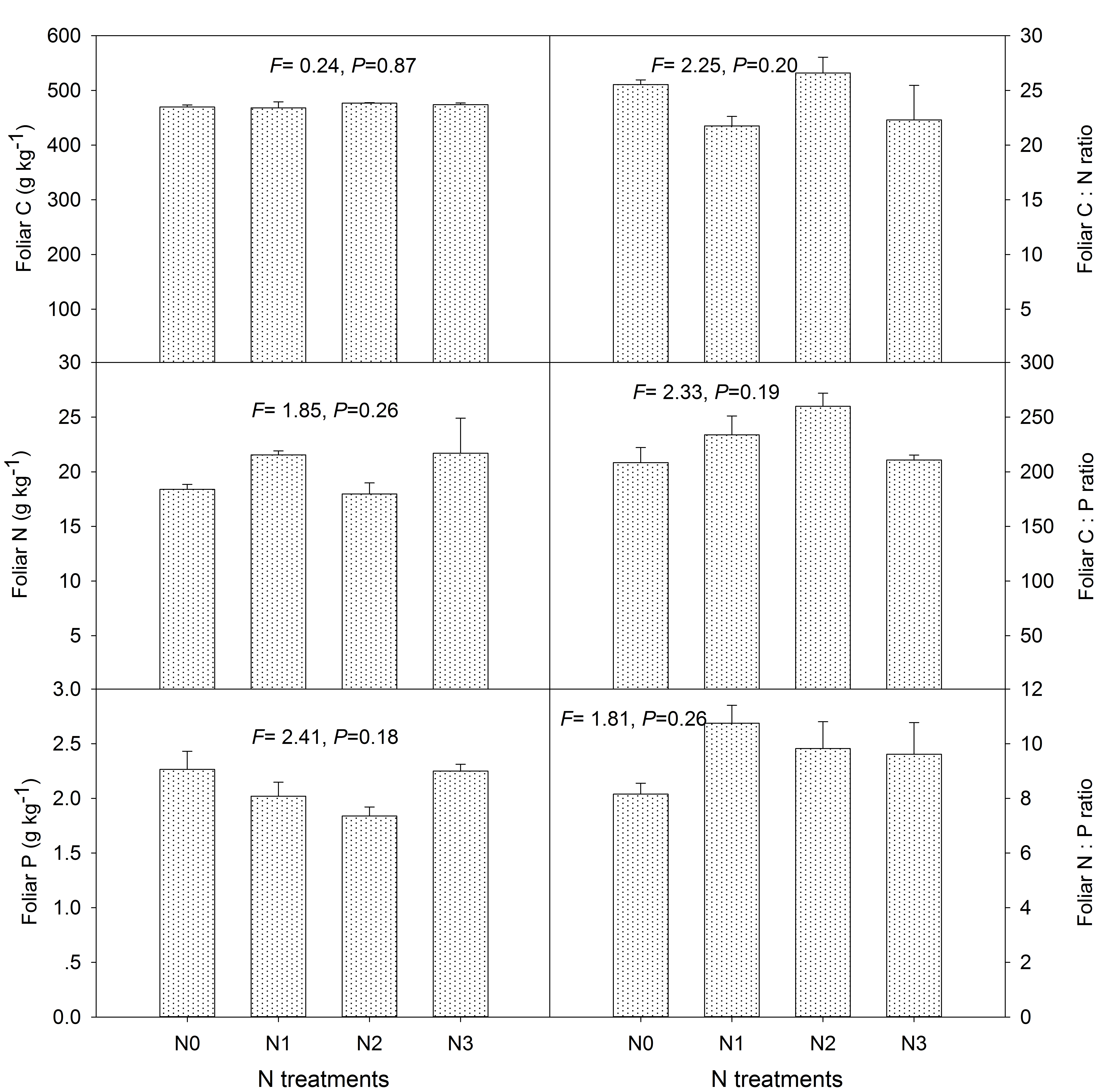


**Figure S1**

**
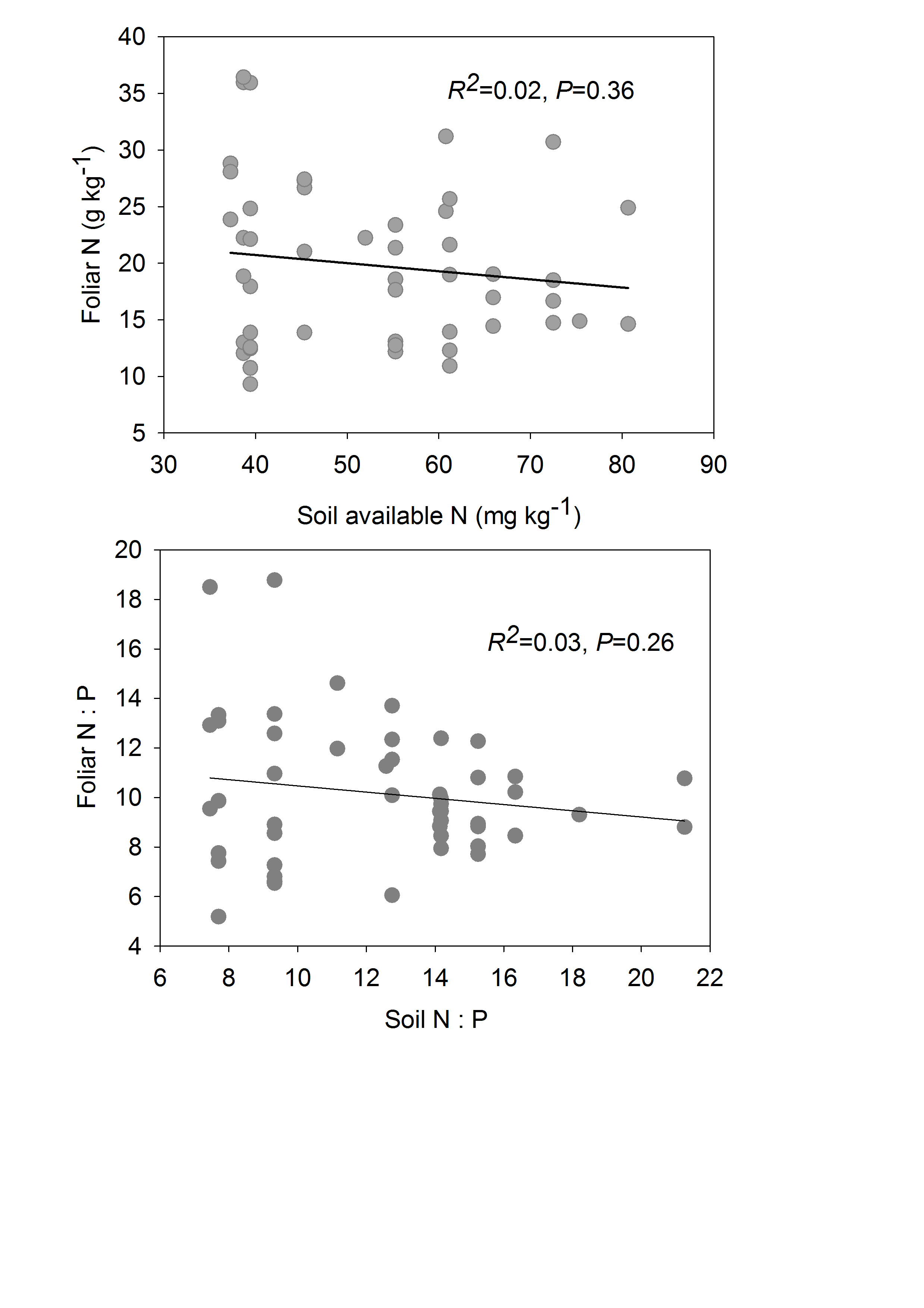
**

**Figure S2**


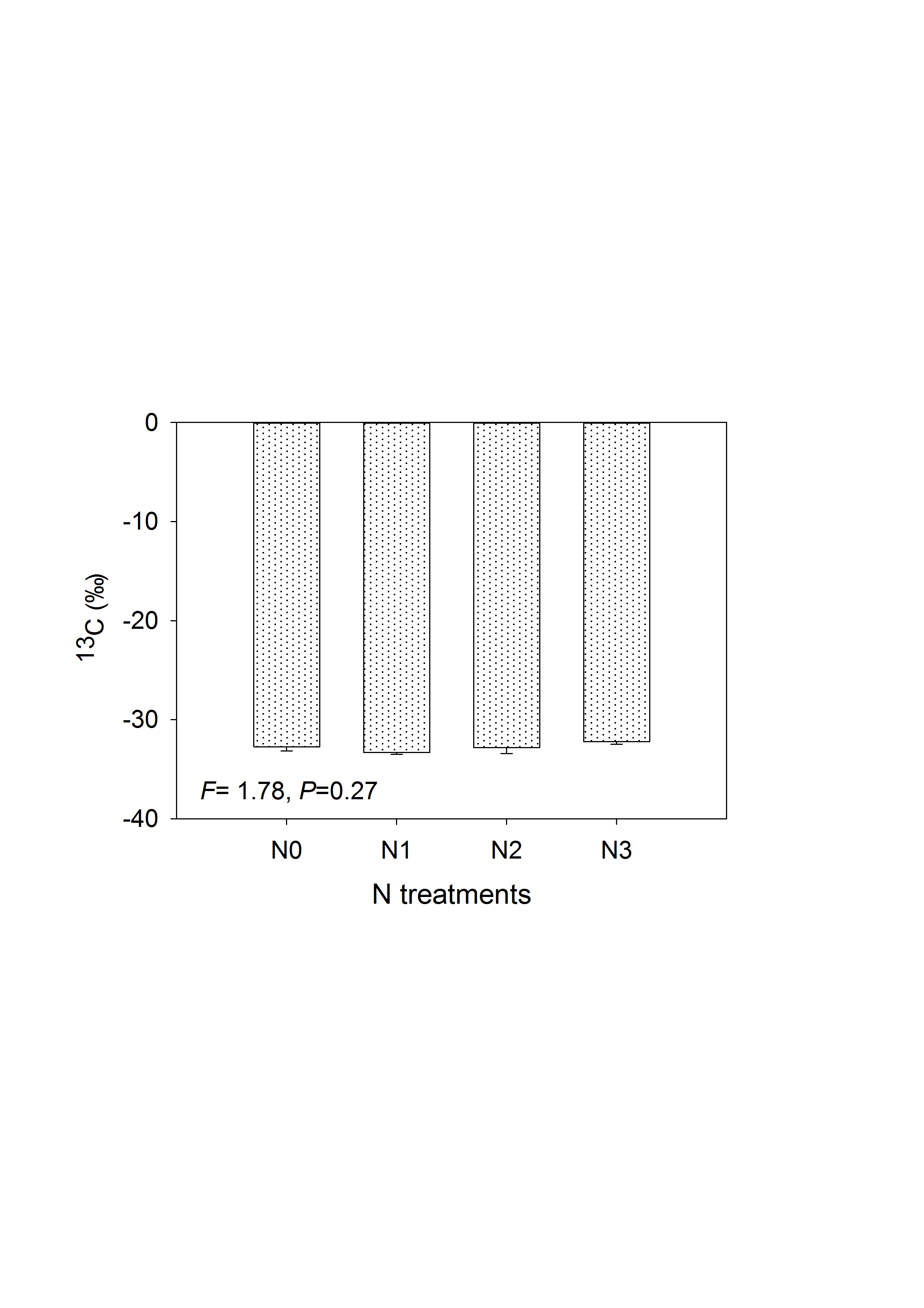


**Figure S3**
